# Quantitative Evaluation of Intraventricular Delivery of Therapeutic Neural Stem Cells to Orthotopic Glioma

**DOI:** 10.3389/fonc.2019.00068

**Published:** 2019-02-19

**Authors:** Margarita Gutova, Linda Flores, Vikram Adhikarla, Lusine Tsaturyan, Revathiswari Tirughana, Soraya Aramburo, Marianne Metz, Joanna Gonzaga, Alexander Annala, Timothy W. Synold, Jana Portnow, Russell C. Rockne, Karen S. Aboody

**Affiliations:** ^1^Department of Developmental and Stem Cell Biology, Beckman Research Institute of City of Hope, Duarte, CA, United States; ^2^Division of Mathematical Oncology, Beckman Research Institute of City of Hope, Duarte, CA, United States; ^3^Department of Cancer Biology, Beckman Research Institute of City of Hope, Duarte, CA, United States; ^4^Department of Medical Oncology & Therapeutics, Beckman Research Institute of City of Hope, Duarte, CA, United States

**Keywords:** glioma, neural stem cells, NSCs, intraventricular administration, therapeutic, drug delivery

## Abstract

Neural stem cells (NSCs) are inherently tumor-tropic, which allows them to migrate through normal tissue and selectively localize to invasive tumor sites in the brain. We have engineered a clonal, immortalized allogeneic NSC line (HB1.F3.CD21; CD-NSCs) that maintains its stem-like properties, a normal karyotype and is HLA Class II negative. It is genetically and functionally stable over time and multiple passages, and has demonstrated safety in phase I glioma trials. These properties enable the production of an “off-the-shelf” therapy that can be readily available for patient treatment. There are multiple factors contributing to stem cell tumor-tropism, and much remains to be elucidated. The route of NSC delivery and the distribution of NSCs at tumor sites are key factors in the development of effective cell-based therapies. Stem cells can be engineered to deliver and/or produce many different therapeutic agents, including prodrug activating enzymes (which locally convert systemically administered prodrugs to active chemotherapeutic agents); oncolytic viruses; tumor-targeted antibodies; therapeutic nanoparticles; and extracellular vesicles that contain therapeutic oligonucleotides. By targeting these therapeutics selectively to tumor foci, we aim to minimize toxicity to normal tissues and maximize therapeutic benefits. In this manuscript, we demonstrate that NSCs administered via intracerebral/ventricular (IVEN) routes can migrate efficiently toward single or multiple tumor foci. IVEN delivery will enable repeat administrations for patients through an Ommaya reservoir, potentially resulting in improved therapeutic outcomes. In our preclinical studies using various glioma lines, we have quantified NSC migration and distribution in mouse brains and have found robust migration of our clinically relevant HB1.F3.CD21 NSC line toward invasive tumor foci, irrespective of their origin. These results establish proof-of-concept and demonstrate the potential of developing a multitude of therapeutic options using modified NSCs.

## Introduction

Despite aggressive surgery, radiation, and chemotherapy, gliomas remain virtually incurable, with median overall survival of patients with glioblastoma, the most common type of malignant glioma in adults, still measured only in terms of months (1–3). The blood-brain barrier (BBB) imposes a major limitation on the delivery of anti-cancer drugs to treat glioma. Glioma cells disseminate from the primary site to form micro-tumor foci throughout the brain, which often “hide behind” the BBB, through which most chemotherapy agents cannot pass (4). The diffuse and highly infiltrative nature of glioma cells further impedes the success of treating gliomas, as no clear border exists between tumor and normal brain tissue, rendering surgical cures elusive.

Human neural stem cell (NSC)-based therapies have emerged as promising strategies for the treatment of central nervous system (CNS) diseases and injury (5–8). Most current clinical trials aim to use NSCs for regenerative purposes: to replace damaged tissue, stimulate repair, or restore missing enzymes. Our NSC-based anti-cancer strategy, however, harnesses the intrinsic tumor-tropic properties of NSCs (9–15), which permit their use as delivery vehicles to selectively target therapeutic gene products to invasive brain tumor cells (16). By modifying NSCs to express a prodrug-converting enzyme, we can potentially produce higher concentrations of chemotherapy drugs directly at tumor sites while minimizing toxicity to normal regions of the brain (17–21).

We demonstrated the safety of a first-generation NSC-mediated gene therapy that utilized a clonal human NSC line genetically modified to express cytosine deaminase (HB1.F3.CD21; CD-NSCs) in a first-in-human study for recurrent glioma patients (19, 21). Cytosine deaminase is an enzyme that converts the orally administered prodrug fluorocytosine (5-FC) to the chemotherapy agent 5-fluorouracil (5-FU). Results from our study included initial demonstration of safety, non-immunogenicity, and proof-of-concept for brain tumor-localized NSC-mediated 5-FU production (21). We also developed a second-generation NSC-mediated enzyme/prodrug gene therapy by adenovirally transducing CD-NSCs to transiently secrete a highly active modified form of human carboxylesterase (hCE1m6) (22). Carboxylesterase (CE) converts the chemotherapy drug irinotecan (IRN) to the 1,000× more potent topoisomerase-1 inhibitor SN-38. We demonstrated that the CE-secreting NSCs (CE-NSCs) are 70-fold more efficient at converting IRN to SN-38 compared to endogenous hCE1 (<5% conversion in the liver and intestines) (23–25).

Intravenously administered IRN has only modest anti-tumor activity in patients with high-grade gliomas (26–28), likely due to poor CNS penetration of its 1,000-fold more active form, SN-38. Our preclinical data in mice bearing orthotopic human glioma demonstrated that after intracerebral/tumoral (ICT) administration, CE-NSCs migrate to distant tumor sites in the contralateral brain (4, 13). *In vivo* pharmacology studies revealed CE-NSC mediated conversion of IRN to SN-38, resulting in concentrations of SN-38 at the tumor site that are 8–10 times higher than concentrations after treatment with IRN alone (22). Treatment with CE-NSCs and IRN significantly extended the survival of human glioma-bearing mice relative to treatment with IRN alone or no treatment (17). Based on these preclinical data, a phase 1 study (clinicaltrials.gov ID **NCT02192359**) is being conducted at City of Hope in patients with recurrent high-grade gliomas using ICT administration to determine the safety and feasibility of ICT administration of CE-NSCs via a Rickham reservoir/catheter system every 2 weeks, followed by intravenous IRN 2 days later.

**Figure 1:**
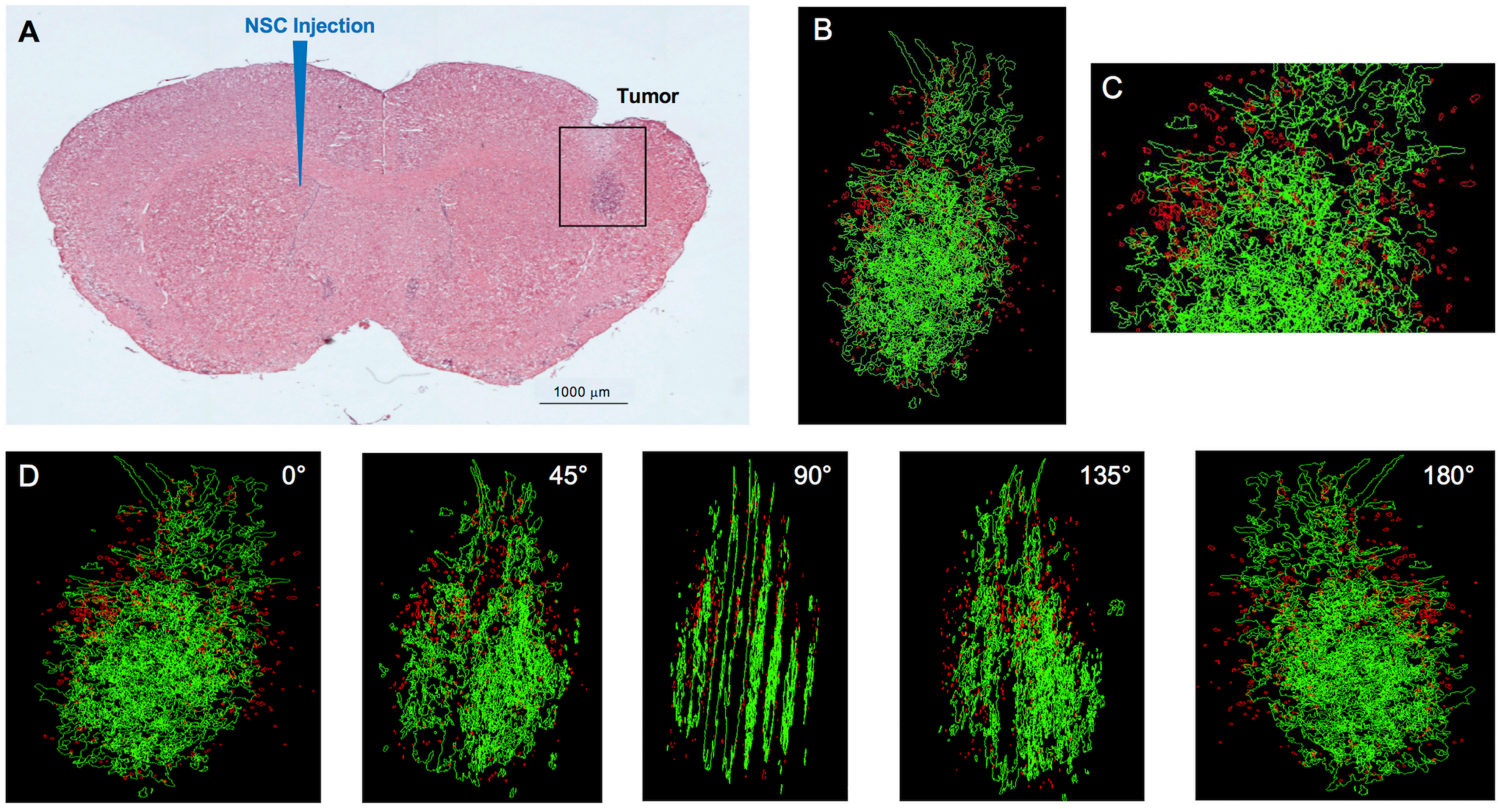
IVEN hCE1m6-NSC distribution in U251 glioma xenografts in *Es1*^*e*^/SCID mice. U251T.eGPF.FFluc tumor cells (2 × 10^5^/2 μl) were injected into the right frontal lobes of *Es1*^*e*^/SCID mice (*n* = 4). At day 10, DiI-labeled CE-NSCs (1.5 × 10^5^/2 μl) were administered into the left ventricle. Brains were harvested 3 days after NSC administration, cryosectioned, and stained with Prussian blue to identify NSCs. **(A)** HE-stained brain tissue section (10 μm) with tumor sites on the right and IVEN NSC injection on the left. Scale bar = 1 mm. **(B)** High-power image (scale bar = 0.2 mm) and **(C)** 3D reconstruction of a U251.eGPF.FFluc tumor xenograft (green) and CE-NSCs (red, pseudo-colored) in the right frontal lobe of an *Es1*^*e*^/SCID mouse. **(D)** Panels show key still images at various rotations. Scale bars have not been provided with the 3D rendered images due to the distortion associated with viewing 3D image projections at different angles when viewed as a 2D image. For reference, the width of the tumor is roughly 300 μm.

IVEN delivery offers five major advantages over ICT delivery: (1) the ability to dose escalate NSCs beyond volume restrictions for ICT administration; (2) improved NSC viability in cerebrospinal fluid (CSF) vs. the hostile environment of the resection cavity; (3) no intratumorally placed catheter tips around which gliosis and scar formation may occur to restrict NSC migration; (4) improved feasibility of performing multi-center studies due to general familiarity with placing Ommaya reservoirs IVEN and using them to administer chemotherapy intrathecally; and (5) potential for CE-NSC mediated gene therapy for treating leptomeningeal metastases from primary and metastatic brain tumors. In this report, we demonstrate that after **intracerebral/ventricular (IVEN)** administration, therapeutic CE-NSCs can migrate to tumors in the brains of mice in three different glioma models: (1) U251 glioma-bearing tumors, (2) patient-derived glioma xenografts (PDXs), and (3) mouse GL261 glioma model (Figure 1). Our data demonstrates the distribution of the CE-NSCs to multiple orthotopic glioma sites in mice following **IVEN administration** (Figure 1).

## Materials and Methods

### Cell Culture

For all studies, we used the v-*myc*-immortalized, human clonal HB1.F3.CD21 NSC line, which is genetically and functionally stable, non-tumorigenic, and minimally immunogenic (19, 29, 30). Briefly, NSCs were thawed and cultured in Dulbecco's modified Eagle's medium (DMEM) supplemented with 10% heat-inactivated fetal bovine serum and 2 mM L-glutamine for 3 days (37°C, 6% CO_2_) in T-175 tissue culture flasks prior to adenoviral transduction, as previously described (22). NSCs were further engineered for high transient expression of a modified human CE (hCE1m6) by transduction with a replication-deficient adenoviral construct.

### *In vivo* Animal Studies

All animal studies were conducted under a protocol approved by the City of Hope Institutional Animal Care and Use Committee (IACUC #04011). Male and female CE-deficient/severe combined immunodeficiency (*Es1*^*e*^/SCID), athymic nude, or C57BL/6 mice (8–12 weeks old) were injected with 2 × 10^5^/2 μl U251T.eGFP.FFluc human glioma cells (U251T; *n* = 6); 2 × 10^5^/2 μl patient-derived PBT017.eGFP.FFluc glioma cells passaged in a mouse brain (PBT017; *n* = 6); or 5 × 10^3^/2 μl GL261 mouse glioma cells (*n* = 5) into the right (U251T and GL261) or both frontal lobes (PBT017). Tumor cells were injected at three different depths 2.25, 2.00, and 1.75 mm. At day 10, post U251 tumor implantation, 2 μl of bolus injection of 4 x10^5^ CE-NSC DiI labeled cells were injected into the left lateral ventricle (+9.0 mm left and −0.3 caudal from bregma) at a depth 2.5 mm. PBT017 (day 14) and GL261 (day 7) tumor bearing mice were given the bolus injection (IVEN) of CE-NSC Molday ion rhodamine B labeled cell at a concentration of 4 × 10^5^ per 2 μl (PBT017) and 2 × 10^5^ per 2 μl (GL261) using same coordinates.

CE-NSCs (4 × 10^5^ cells/2 μl) labeled with Molday ION Rhodamine B were administered into the left lateral ventricle on day 10 post U251 implantation; day 14 post PBT017 implantation; or day 7 post GL261 implantation (each tumor latency and engraftment time was previously determined: Aboody et al., unpublished data). Mice were monitored daily for distress and discomfort in accordance with the recommendations of the Panel of Euthanasia of the American Veterinary Medical Association. Euthanasia was conducted on day 3 after CE-NSCs administration in a CO_2_ chamber that enabled visualization of the animals to minimize distress during euthanasia with a gradual increase in the flow of CO_2_.

### Histopathology and Staining

Brains were fixed in 4% paraformaldehyde (PFA) for 72 h and transferred to 70% EtOH (or 30% sucrose) solution for dehydration for 3–5 days. Frozen brain sections were prepared (10 μm) and every 10th section was stained with hematoxylin eosin (HE) to detect the tumor and Prussian blue staining using an Accustain Iron Stain Kit (Sigma-Aldrich) to identify the presence of CE-NSCs. Tumors and CE-NSCs were visualized by bright field imaging.

### 3D Reconstruction

Three-dimensional reconstruction was performed using Reconstruct software (SynapseWeb, version 1.1). 9–15 images of serial 10 μm H&E and Prussian blue-stained brain sections were imported into Reconstruct and aligned manually. To produce a 3D image, structures of interest were segmented based on color (Prussian blue for NSCs) and cell density (HE to highlight tumor areas), as described previously (13).

### Analysis of CE-NSC Spatial Distribution

CE-NSCs in the mouse brains were identified and quantified to elucidate the patterns of spatial distribution in the brain, especially around the tumors. CE-NSCs stained in Prussian blue were identified on each of the IHC stained slices using the open-source image processing software ImageJ (31). The centers of the clusters of CE-NSCs identified using “color thresholding” and “analyze particles” tools were tabulated. A tumor mask was generated by manually delineating the edges of the tumor using the “polygon selection” tool. The center of this mask was identified as the tumor center of mass and the distance and orientation of the CE-NSC clusters with respect to the tumor center was calculated. The results for all the IHC slices were combined to generate a polar histogram in MATLAB 2018a (Mathworks, Natick, MA) representing the spatial distribution of CE-NSCs with respect to the tumor center for each mouse brain. The size of the bars in the plots indicates the percentage of NSCs found along that radial direction, and the color of the bars indicates the distance from the tumor center. Because mice implanted with the PBT017 cell line were injected with dual tumors, CE-NSCs identified in the left hemisphere were associated with the tumor in the left hemisphere and CE-NSCs identified in the right hemisphere were associated with the tumor in the right hemisphere. Thus, 2 polar histograms were generated for each mouse.

## Results

### Migration and Localization of IVEN-Administered CE-NSCs to Brain Tumor Sites *in vivo*

**Figure 2:**
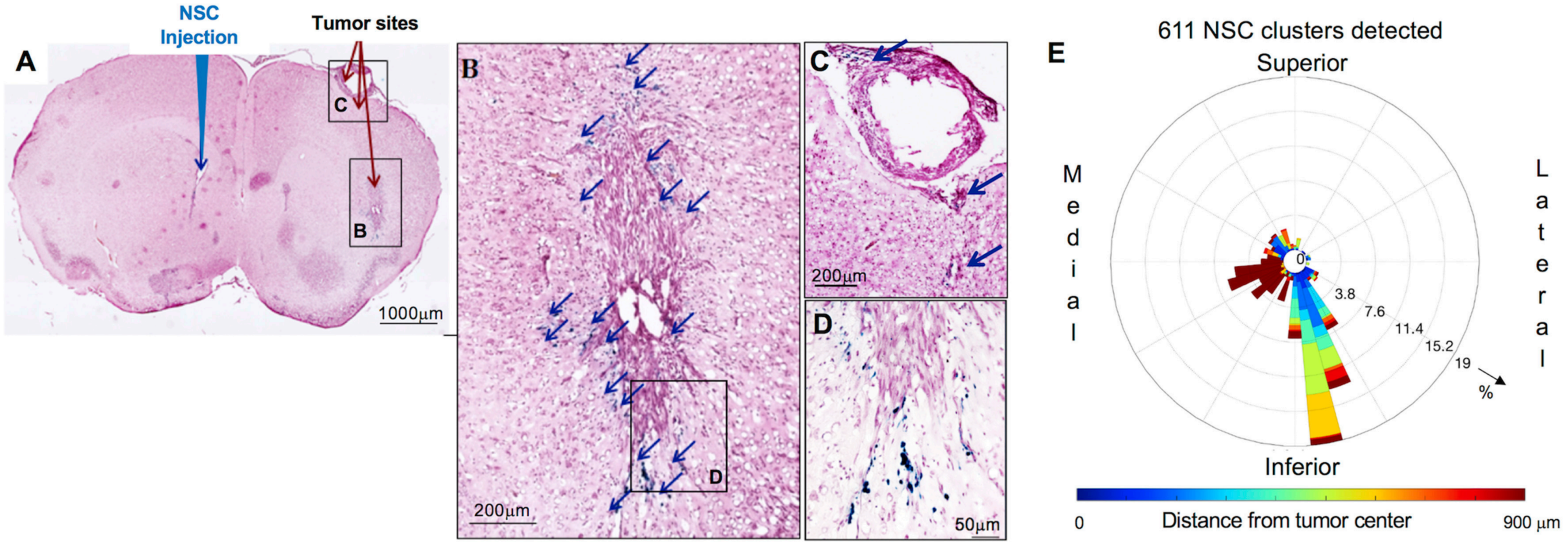
Distribution and spatial analysis of IVEN hCE1m6-NSC migration to U251 glioma xenografts in athymic nude mice. U251T.eGPF.FFluc tumor cells (2 × 10^5^/2 μl) were injected into the right frontal lobe of athymic nude mice (*n* = 4). At day 10, Molday-labeled CE-NSCs (4.0 × 10^5^/2 μl) were injected into the left ventricle. Brains were harvested 3 days after NSC.CD administration, cryosectioned, and stained with Prussian blue to identify NSCs. **(A)** HE-stained brain tissue section (10 μm) with tumor sites on the right and IVEN NSC injection on the left (scale bar is 1 mm). **(B,C,D)** Insets from **(A)**: magnified images of Prussian blue-stained NSCs, indicated with blue arrows (scale bars 200, 200, 50 μm, respectively). **(E)** Polar histogram of CE-NSC distribution demonstrates the spatial distribution of NSC clusters around the tumor center. CE-NSCs close to the tumor (blue bars) were found along the superior-medial, inferior, and inferior-lateral directions of the tumor. NSCs found far away from the tumor (red bars) were found in the contralateral ventricle.

To initiate a xenograft model of glioma, adult *Es1*^*e*^/SCID immunodeficient mice were implanted with U251T.eGFP.FFluc cells into the right frontal lobe. On day 10, Molday-labeled CE-NSCs were administered into the left lateral ventricle. When injected IVEN into U251T glioma-bearing mice, CE-NSCs migrated to tumor xenografts established in the opposite hemisphere (Figures 1, 2). These CE-NSCs were visualized in the vicinity of the tumor and were not detected in non-tumor brain parenchyma.

The distribution of Molday-labeled CE-NSCs to U251T tumors was also quantitated in athymic nude mice. CE-NSCs were visualized by Prussian blue staining (Figures 2A–D) and migration was quantitated by polar histogram (Figure 2E). The polar histogram shows the quantified spatial distribution of CE-NSCs around the tumor, including both the number of CE-NSCs in various directions and their distance from the tumor center. CE-NSCs demonstrated preferential localization around the tumor, with the CE-NSCs closest to the tumor along the superior-medial and inferior sides. CE-NSCs along the medial direction and far from the tumor represent those in the contralateral ventricle.

### Migration and Localization of CE-NSCs to Bilateral PDX Tumor Sites Following Injection Into the Left Ventricle

**Figure 3:**
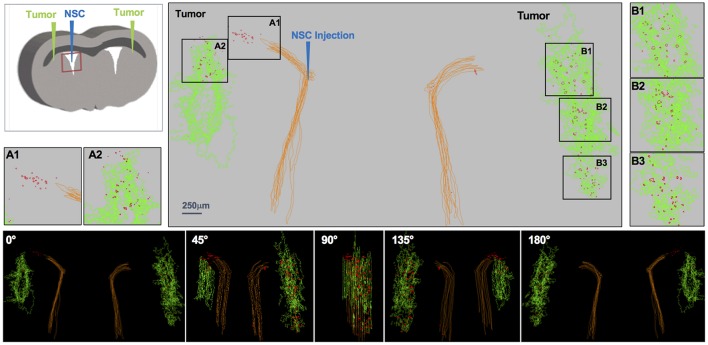
IVEN hCE1m6-NSC distribution in PDX glioma tumors in *Es1*^*e*^/SCID mice. PBT017.eGFP.FFluc cells (2 × 10^5^/2 μl) were injected into the right and left frontal lobes of *Es1*^*e*^/SCID mice (*n* = 6). At day 14, Molday-labeled CE-NSCs (4.0 × 10^5^/2 μl) were administered into the left lateral ventricle. Brains were harvested and histological sections (10 μm) prepared on day 17. Every 10th section was stained with HE to visualize the tumors and Prussian blue to visualize the NSCs. 3D reconstructions of the right and left PBT017 tumors (green) with right and left lateral ventricles (brown) are shown. Insets A1 and A2 demonstrate CE-NSCs (red) migrating toward left tumor; B1–B3: NSCs migrating to right tumor foci. Also shown are the tumor and the NSCs visualized from different viewing angles.

**Figure 4:**
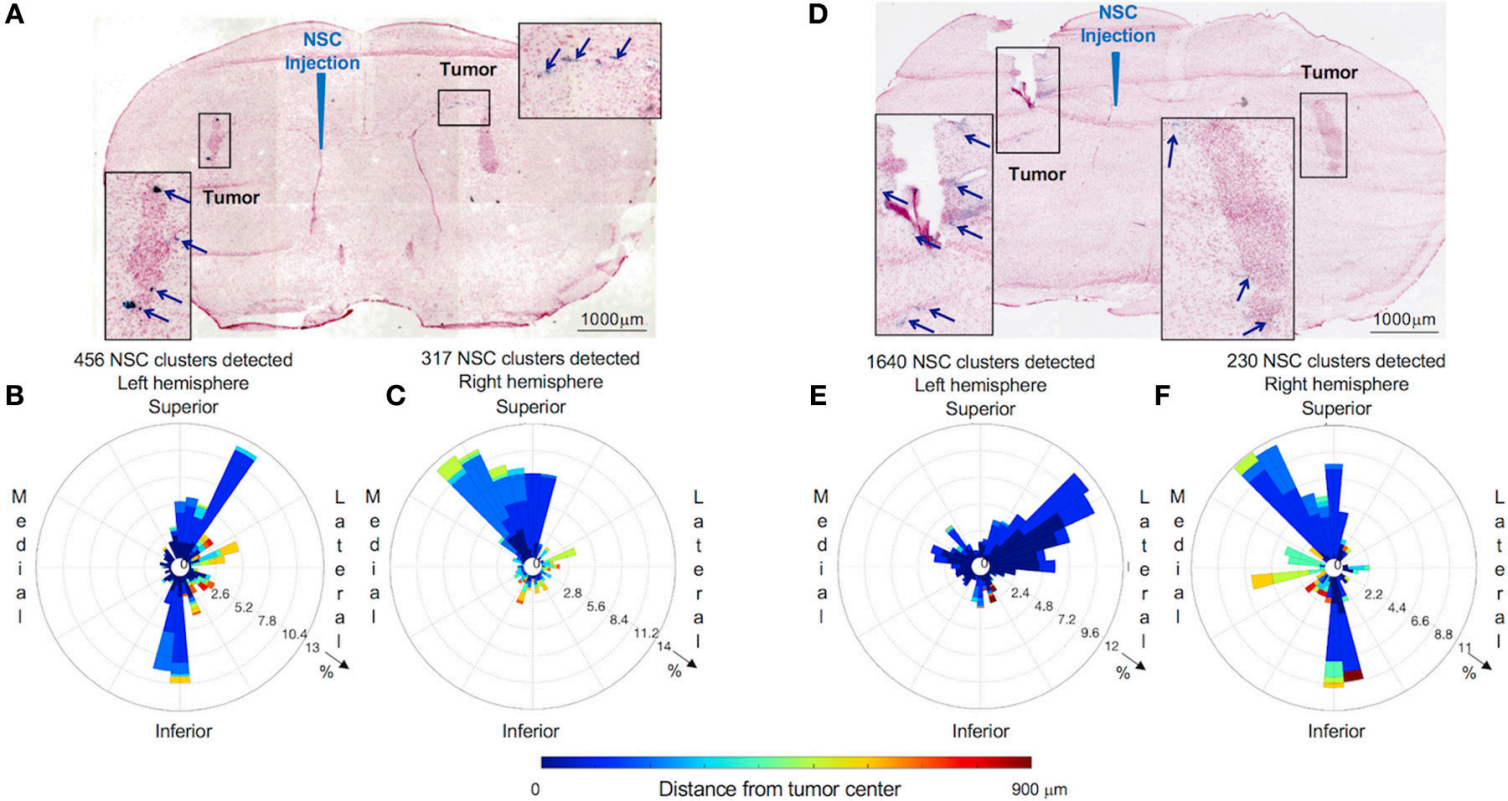
Distribution and spatial analysis of IVEN hCE1m6-NSC migration to bilateral PDX glioma tumors in *Es1*^*e*^/SCID mice. PBT017.eGFP.FFluc cells (2 × 10^5^/2 μl) were injected into the right and left frontal lobes of *Es1*^*e*^/SCID mice (*n* = 6). At day 14, Molday-labeled CE-NSCs (4.0 × 10^5^/2 μl) were administered into the left lateral ventricle. Brains were harvested, cryosectioned, and stained with Prussian blue to identify NSCs. **(A)** Histological section (10 μm) stained with Prussian blue to identify CE-NSCs (scale bars 1,000 μm). Insets show the localization of CE-NSCs near tumors in the left and right hemispheres of the mouse brain. Polar histograms of CE-NSCs identified in the **(B)** left and **(C)** right hemispheres are shown. **(D,E,F)** Same as **(A,B,C)** for a different mouse. Polar histograms show the majority of identified CE-NSCs within 200 μm of the tumor, demonstrating the capability of the cells to migrate to the tumor. Additionally, these data show preferential accumulation of CE-NSCs along the superior-medial and inferior directions of the tumors. Notably, the number of identified CE-NSC clusters were similar in the left and right hemispheres of the first mouse. In the second mouse, CE-NSCs were preferentially found in the left hemisphere.

To analyze migration of CE-NSCs to multiple tumor foci within brain parenchyma after IVEN administration, dual tumors were initiated in *Es1*^*e*^/SCID mice via bilateral administration of human patient-derived glioma cells PBT017. Fourteen days later, CE-NSCs were administered into the left ventricle, after which the CE-NSCs migrated to both left and right tumor sites (Figure 3). Migration of the CE-NSCs was analyzed by 3D reconstruction (Figure 3) and polar histogram analysis (Figure 4). Since a tumor was inoculated in each hemisphere, a polar histogram was generated for each tumor with CE-NSCs identified in any given hemisphere attributed to the tumor present in that hemisphere. The histological sections show substantial CE-NSC presence around the tumors (Figures 4A,D). This observation is reflected in the polar histograms (Figures 4B,C,E,F) with nearly all the bars in blue indicating CE-NSCs in the proximity of the tumor. Strong signals indicate preferential localization of CE-NSCs along the superior-medial and inferior directions, indicating possible migration of CE-NSCs into the tumor along these directions. Notably, one mouse (Figure 4A) was observed to contain equivalent distributions of CE-NSCs in both hemispheres, whereas another mouse (Figure 4D) was observed to contain a significantly higher distribution of CE-NSCs in the tumor in the left (ipsilateral to CE-NSC injection site) hemisphere.

### Migration and Localization of CE-NSCs to Gl261 Murine Glioma Tumor in C57BL/6 Mice

**Figure 5:**
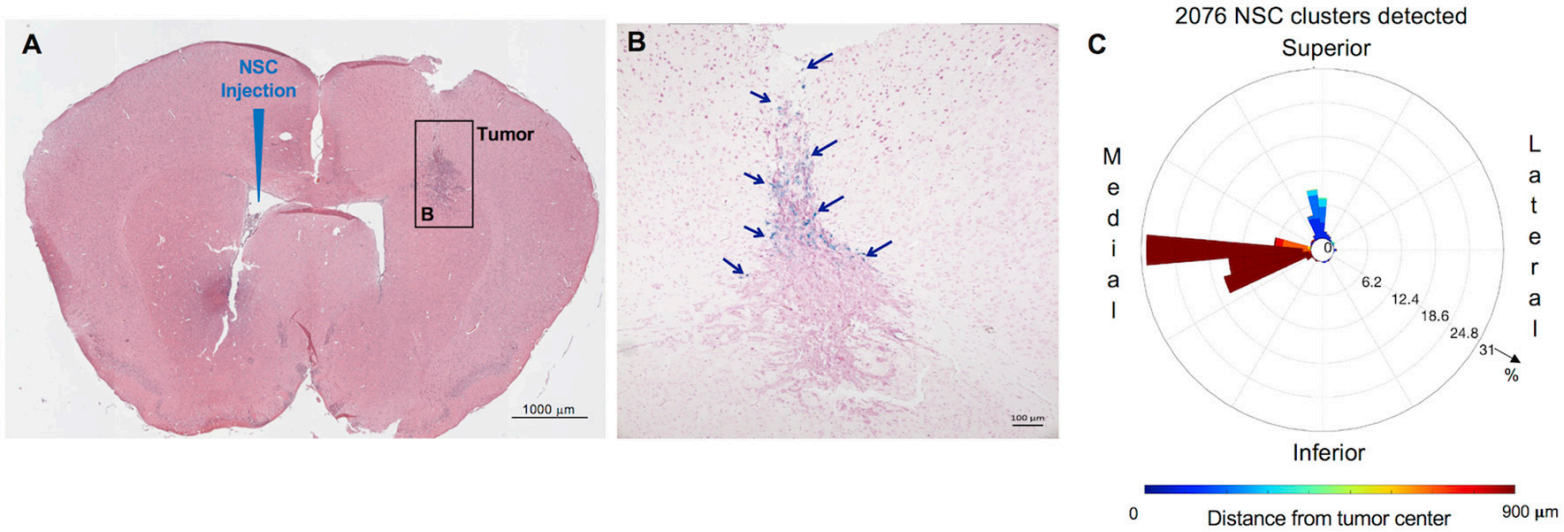
Distribution and spatial analysis of IVEN hCE1m6-NSC migration to GL261 murine glioma tumors in C57BL/6 mice. GL261 tumor cells (5 × 10^3^/2 μl) were in injected into the right frontal lobe of C57BL/6 mice (*n* = 5). At day 7, Molday-labeled CE-NSCs (2.0 × 10^5^/2 μl) were injected into the left ventricle. Brains were harvested 2 days after CE-NSC administration, cryosectioned, and stained with Prussian blue to identify NSCs. **(A**,**B)** HE-stained brain tissue section (10 μm) with tumor sites on the right and IVEN NSC injection on the left (scale bars 1,000 and 100 μm, respectively). **(C)** Polar histogram of NSC distribution around the tumor. NSCs can be observed to invade the tumor from the superior direction (blue bars), consistent with NSCs visualized in the tumor. Distant clumping of the CE-NSCs in the contralateral ventricle can also be observed (red bars seen medially).

C57BL/6 mice were injected with GL261 cells (5 × 10^3^ cells/2 μl) into the right frontal lobe, as described above. On day 7 of the study, CE-NSCs were injected into left lateral ventricle. CE-NSCs demonstrated robust migration from the ventricles into the tumors. The histological section presented in Figure 5B exhibits what appears to be active migration of CE-NSCs from the ventricles and partially from the subarachnoid space/tumor administration needle track. The invasion of the tumor by CE-NSCs from the superior end is also reflected by the blue bar in the polar histogram (Figure 5C) toward the superior direction. CE-NSCs along the medial direction and far from the tumor represent those in the contralateral ventricle. Aggregation of CE-NSCs in the contralateral ventricle was observed.

## Discussion

Innately tumor-tropic NSCs are able to penetrate the BBB and migrate through brain parenchyma to efficiently localize to both the primary brain tumor site and invasive foci that often seed recurrent disease. These features provide an unprecedented opportunity to develop an effective, tumor-selective therapy for patients with malignant brain tumors. NSCs can produce increased concentrations of tumor-localized chemotherapy while minimizing toxicity to normal brain tissue. We have demonstrated the efficacy of ICT-administered CE-NSCs + IRN in preclinical studies; however, there are multiple drawbacks to ICT administration of NSCs to brain tumor patients, as described above. Our data demonstrate that IVEN administration of NSCs results in similar distribution and tumor coverage in orthotopic tumors that are both proximal and contralateral to the site of injection. By administering NSCs IVEN rather than ICT, we can optimize therapeutic dosing and potentially increase distribution to tumor sites throughout the brain. Specifically, the major advantages include: (1) the ability to dose escalate NSCs beyond volume restrictions for ICT administration; (2) improved NSC viability in CSF; (3) no intratumorally placed catheter tips; (4) improved feasibility of performing multi-center studies; and (5) potential for CE-NSC-mediated gene therapy for treating leptomeningeal metastases from primary and metastatic brain tumors. Beyond the current NSC-based enzyme/prodrug converting strategies to increase levels of cytotoxic chemotherapy in brain tumors, we envision using our tumor-tropic NSCs as a platform technology can be further modified for tumor-localized delivery of a variety of anti-tumor products, such as apoptotic agents, oncolytic viruses, and antibodies, which could potentially be administered serially or in combination to maximize therapeutic benefit (32). Therefore, the impact of optimizing the delivery of NSCs may be far-reaching.

It should be noted that several challenges remain for therapeutic optimization. This includes determination of dosing and regimen that results in maximal tumor coverage, and more uniform distribution through each tumor mass. Multiple and complex factors can affect NSC tumor tropism including tumor-derived growth factors hepatoxcyte growth factor (HGF), endothelial growth factor (EGF), vascular endothelial growth factor (VEGF), urokinase plasminogen activator (uPA), extracellular matrices (ECM), stromal cell-derived factor 1 (SDF-1), hypoxia inducible factor (HIF-1α, and inflammatory cytokines (e.g., IL-6 and IL-8) (15). Thus, tumor size, location, and heterogeneity likely contribute to the non-uniformity of NSC distribution within a given tumor mass. Clinical correlative studies that include more refined and sophisticated imaging analysis, in addition to intracerebral microdialysis and histopathology, may shed more light on this subject.

In our first-in-human study, we documented NSC migration to distant tumor foci in the human brain at the time of autopsy. Permission for brain autopsy was obtained from the families of two study participants. The autopsied brains were extensively sampled, including areas adjacent to and distant from the CD-NSC injection site and ipsilateral and contralateral areas of obvious tumor involvement, as well as deep nuclei, periventricular areas, long axonal tracts, cortical gray matter, and subcortical white matter. All of the samples areas were assessed for the presence of CD-NSCs by nested PCR for the v-*myc* gene. In both brains, v-*myc*-positive areas of single cells were detected distant from the primary injection sites (including the opposite hemisphere) in areas of tumor cells.

We observed aggregation of CE-NSCs within the left ventricle (injection site) of a mouse bearing GL261 murine glioma cells (Figure 5). Although the migration of CE-NSCs to the tumor site was evident, such aggregation of CE-NSCs at the injection site might result in a loss of therapeutic efficiency. We suspect that the aggregation was caused by rapid injection of the CE-NSCs. Thus, the rate and number of NSCs injected must be adjusted and properly monitored to achieve optimal therapeutic efficiency. However, human ventricles are much larger than mouse ventricles; therefore, we do not expect such clumping when CE-NSCs are used as therapeutics clinically.

We observed that CE-NSCs localized along the superior-medial and inferior directions around the tumors established from U251 and PBT017 cell lines. The superior-medial localization of CE-NSCs can be attributed to invasion of CE-NSCs from the ventricle into the tumor. In mice bearing GL261 tumors, CE-NSCs localized along the superior direction around the tumors, indicating invasion from the subarachnoid space along the tumor cell injection track. We previously demonstrated migration of NSCs along white matter tracts (33). However, it was also documented that the NSCs migrated with CSF flow through the third and fourth ventricles and subarachnoid space and entered the tumor site through the tumor injection needle track, consistent with our observations. The utilization of such diverse migration routes to tumor sites strongly supports the use of IVEN-delivered CE-NSCs as delivery vehicles for a variety of anti-cancer therapeutics. A potential limitation is the immune mediated impact of delivery of NSCs via IVEN, this will need to be carefully assessed prior to translation to the clinic.

## Data Availability

All datasets for this study are included in the manuscript files. The raw data supporting the conclusions of this manuscript will be made available by the authors to any qualified researcher.

## Author Contributions

LF, LT, RT, SA, MM, JG, and AA performed experiments. VA performed quantitative analysis and produced figures. RR and VA designed the quantitative analyses. AA, TS, and JP were involved in discussion and data analysis. MG, KA, and RR led the project, contributed to experimental design, analyses design, review and discussion. All authors reviewed the final manuscript.

### Conflict of Interest Statement

KA and AA are founders and shareholders of Therabiologics, Inc. The remaining authors declare that the research was conducted in the absence of any commercial or financial relationships that could be construed as a potential conflict of interest.
